# Comparative Profiling of *Pseudomonas aeruginosa* Strains Reveals Differential Expression of Novel Unique and Conserved Small RNAs

**DOI:** 10.1371/journal.pone.0036553

**Published:** 2012-05-10

**Authors:** Silvia Ferrara, Margherita Brugnoli, Angela De Bonis, Francesco Righetti, Francesco Delvillani, Gianni Dehò, David Horner, Federica Briani, Giovanni Bertoni

**Affiliations:** Dipartimento di Scienze Biomolecolari e Biotecnologie, Università degli Studi di Milano, Milan, Italy; Vrije Universiteit Brussel, Belgium

## Abstract

*Pseudomonas aeruginosa* is a highly adaptable bacterium that thrives in a broad range of ecological niches and can infect multiple hosts as diverse as plants, nematodes and mammals. In humans, it is an important opportunistic pathogen. This wide adaptability correlates with its broad genetic diversity. In this study, we used a deep-sequencing approach to explore the complement of small RNAs (sRNAs) in *P. aeruginosa* as the number of such regulatory molecules previously identified in this organism is relatively low, considering its genome size, phenotypic diversity and adaptability. We have performed a comparative analysis of PAO1 and PA14 strains which share the same host range but differ in virulence, PA14 being considerably more virulent in several model organisms. Altogether, we have identified more than 150 novel candidate sRNAs and validated a third of them by Northern blotting. Interestingly, a number of these novel sRNAs are strain-specific or showed strain-specific expression, strongly suggesting that they could be involved in determining specific phenotypic traits.

## Introduction

Small RNAs (sRNAs) are widespread in bacteria and play critical regulatory roles in several cellular processes [1]–[4]. The prototype of a bacterial sRNA is a non-coding RNA 50-300 nucleotides long that acts by imperfect base pairing with *trans*-encoded RNA target(s). sRNA-target interaction may lead to modulation of mRNA translation and/or stability [2], [4]. Variations on this theme are also known. For instance, some sRNAs modulate the activity of target proteins or act as mRNAs coding for short proteins. Moreover, there is growing evidence that many sRNAs are *cis*-encoded and transcribed antisense to their target RNA [5]. The target genes of sRNAs-mediated regulation belong to several different functional groups. The prevalent view is that sRNAs might target almost all bacterial cell processes [6]. In pathogenic microbes, several sRNAs have been shown to be involved in host-microbe interactions and in the adaptation to the host environment [6]. In recent years, genome-scale searches have led to a remarkable increase in the number of identified sRNAs in bacteria [2]. In this context, our knowledge of the sRNA complement of *Pseudomonas aeruginosa* seemed limited.

*P. aeruginosa* is a highly adaptable bacterium which thrives in a broad range of ecological niches. In addition, it can infect multiple hosts as diverse as plants, nematodes and mammals. In humans, it is an important opportunistic pathogen in compromised individuals, such as patients with cystic fibrosis, severe burns and impaired immunity [7], [8]. The broad habitat and host ranges of *P. aeruginosa* reflect the large variety of structural, metabolic and virulence functions found in its pangenome (being 6.2–6.9 Mbp the size range of the sequenced strain genomes) [9]–[12] composed of a high proportion (approximately 90%) of conserved core genes and a rather small accessory genome, found in some strains but not in others, which includes genetic elements supposed to be acquired by horizontal transfer. Accessory genetic elements can confer specific phenotypes that are advantageous under the selective pressure of given habitat or host conditions [10]. Interestingly, a study on the highly virulent strain PA14 has suggested that pathogenicity requires not only virulence factors encoded in the two pathogenicity islands of the accessory genome, but also several core genes [13]. Thus, there seems to be some combinatorial effects between accessory and core functions. In addition, it seems likely that the coordination of the expression of such a panoply of functions is accomplished by regulatory networks based on a large number of regulators. Strikingly, the genome of the archetypal strain PAO1 was found to contain among the highest proportions (9–10%) of regulatory genes as compared to other sequenced bacterial genomes, there being more than 500 genes predicted to encode either transcriptional regulators or two-component regulatory system proteins [11], [12], [14]. In contrast, only a small number (about 40) of regulatory sRNAs have been reported in *P. aeruginosa* [15] whereas, for example, more than 100 sRNAs have been described in *Escherichia coli* and *Salmonella* [1], [3], [16], whose genomes are considerably smaller than *P. aeruginosa*.

The apparent low proportion of sRNAs in *P. aeruginosa* could reflect either a real paucity of regulatory sRNAs or the limited number of genome-wide searches that have been performed in this species [17]–[19]. In addition, only few of the sRNAs experimentally validated in *P. aeruginosa* have been functionally characterized to date; they have been implicated in carbon catabolite repression (CrcZ) [20], in virulence genes expression control (RsmY,Z) [21]–[23], or in other functions that can be important for survival in the infected host, such as iron uptake and storage (PrrF1) [24] and *quorum sensing* (PhrS) [25]. Finally, despite the variable degree of virulence shown by different *P. aeruginosa* isolates [13], experimental sRNAs screening has been performed only on PAO1. The identification of genes differentially expressed in virulent *vs*. attenuated strains, irrespective of whether they belong to core or accessory genome, can be a valuable approach for dissecting pathogenicity in this bacterium. This would be particularly true for genes encoding regulatory factors, such as sRNAs, whose expression level may in turn influence the expression of multiple target genes.

In this work we aimed at the systematic identification of sRNAs of *P. aeruginosa* by means of the recently developed “sRNA-Seq” approach, an unbiased high-throughput method for the screening of the entire sRNA complement of any organism based on “next-generation” sequencing technologies [26]. We applied the sRNA-Seq method both to PAO1 and to the highly virulent strain PA14, which differ for the presence of about 112 strain-specific gene clusters (54 PAO1-specific and 58 PA14-specific, including the two PA14 pathogenicity islands PAPI-1 and PAPI-2) [13].

By using this approach, we have identified more than 150 novel candidate sRNAs in *P. aeruginosa*. Interestingly, a relevant number of sRNA hits were strain-specific or showed strain-specific expression, strongly suggesting that they could be involved in determining strain-characteristic phenotypic traits. We probed by Northern blotting 71 candidates and confirmed the expression of 52 new sRNAs, with a validation rate above 73%. Our results expand the panel of *P. aeruginosa* sRNAs resulting from previous surveys and strongly indicate that the degree of sRNAs utilization as regulators is consistent with other bacterial species.

## Methods

### RNA Isolation and Generation of sRNA-Seq Amplicon Libraries

Total RNA was prepared from 25 ml samples of early stationary phase (A_600_ of about 2.6) cultures of *P. aeruginosa* strains PAO1 [14] and PA14 [27] grown at 37°C in 100 ml of Brain Heart Infusion (BHI) rich medium in 500-ml flasks vigorously shaken (120 rpm). The cells were recovered by centrifugation, resuspended in RNAprotect Cell Reagent (Qiagen) and incubated for 5 min at room temperature, pelleted by centrifugation and stored at −80°C until use. Cells were resuspended in TE–lysozyme (10 mM Tris HCl, 1 mM EDTA, 1 mg/ml lysozyme, pH 7.5), incubated at room temperature for 5 min, and lysed by QIAzol Lysis Reagent (Qiagen). Total RNA was then extracted by the RNeasy Mini Kits (Qiagen) according to the manufacturer’s instructions, including RNase-free DNase I in-column treatment and modifications to enrich for small RNAs (<200 nt). The quality of the RNA was assessed by denaturing (8 M urea) 6% polyacrylamide gel electrophoresis (dPAGE).

Size selection of RNA ranging from 20 to 500 nt was performed by fractionating 160 µg of total RNA on preparative dPAGE and cutting the gel slice containing 20 to 500 nt long transcripts. RNA from 20 to 500 nt was electroeluted from gel slices in a Model 422 Electro-Eluter (Biorad). For the preparation of amplicon libraries, the purified 20–500 nt RNA fraction of each strain was first tagged at the 3′-end with linker L1 (Table S1), a 5′-monophosphate oligonucleotide starting with three ribonucleotides followed by a sequence of 20 deoxyribonucleotides and terminally protected with an inverted dT (IDT, Integrated DNA Technologies). The sequence of this hybrid oligonucloeotide does not match any sequence in the *P. aeruginosa* genome and is predicted not to form complex secondary structures. 60 µg of RNA was ligated with 78 µg of L1 in T4 RNA ligase Buffer, 10% DMSO, at 16°C with 90 U of T4 RNA ligase (New England Biolabs). After 16 hrs, additional 90 U of T4 RNA ligase was added and incubation prolonged for 8 hrs. To check ligation efficiency, 0.5–1 µg of RNA from the ligation reaction was probed by Northern blotting with [^32^P]-labelled -oligos AL1 and PA5SRNA02 (Table S1), which probe L1 and 5S rRNA, respectively. To remove non-ligated L1, the ligation mixtures were run on preparative dPAGE and RNA ranging from 40 to 520 nt was electroeluted from gel slices as described above.

**Table 1:** nstSGR distribution in PAO1 and PA14.

Loci	nstSGR group	PAO1	PA14
Unique	A	9	−
	B	−	20
Conserved	C	43	−
	D	76	76
	E	−	72
Total	220	128	168

**Figure 1:**
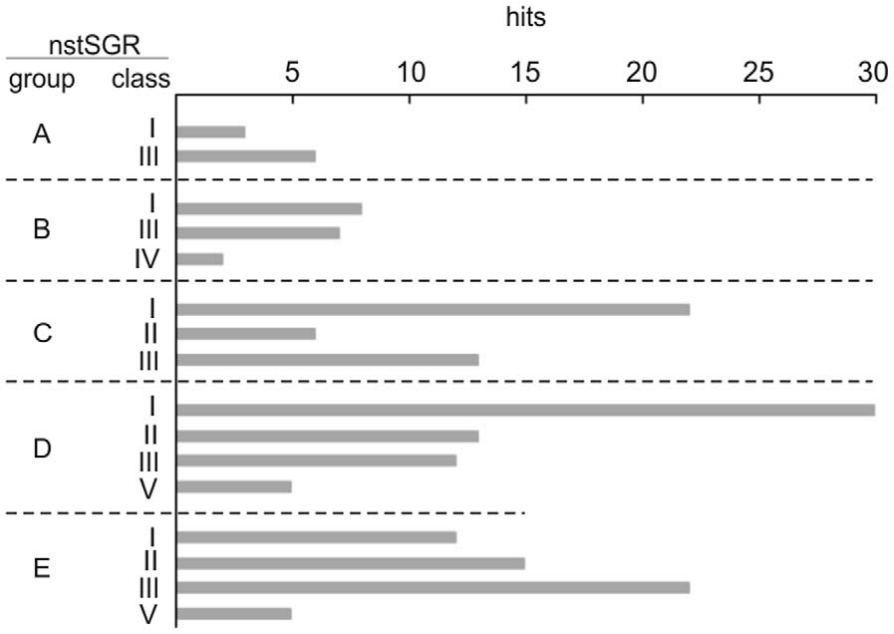
Classes of candidate sRNAs and their distribution within the nstSGR groups resulting from sRNA-Seq. The histogram summarizes the data of Table S2. Candidate sRNAs identified by sRNA-Seq were categorized into five structural/functional classes (I, sRNAs; II, 5′-UTRs; III, asRNAs; IV, CRISPRs; V, sRNAs overlapping annotated ORFs) according to the criteria depicted in Figure S3 and distributed within each nstSGR group (A and B, unique in PAO1 or PA14, respectively; C and E, conserved in both strains but expressed in either PAO1 or PA14, respectively; D, conserved and expressed in both strains).

RNase H depletion of tRNA and 5S rRNA was performed as previously described [26] with some modifications. 30 µg of L1-sRNA_20–500_ was annealed to 9 nmol of Oligo Mix, an equimolar mixture of 47 oligonucleotides (Table S1) complementary to the 3′-ends of *P. aeruginosa* tRNAs and 5S rRNA. The RNA-DNA hybrids were then digested with RNase H so as to remove the 3′-L1 tail from the small stable RNAs. Depletion efficiency was checked by Northern blotting with [^32^P]-labelled oligos AL1 and PA5SRNA02. RNA-L1 was then separated from Oligo Mix, tRNA and 5S rRNA degradation products by preparative dPAGE and electroelution from gel slices as described above.

**Figure 2:**
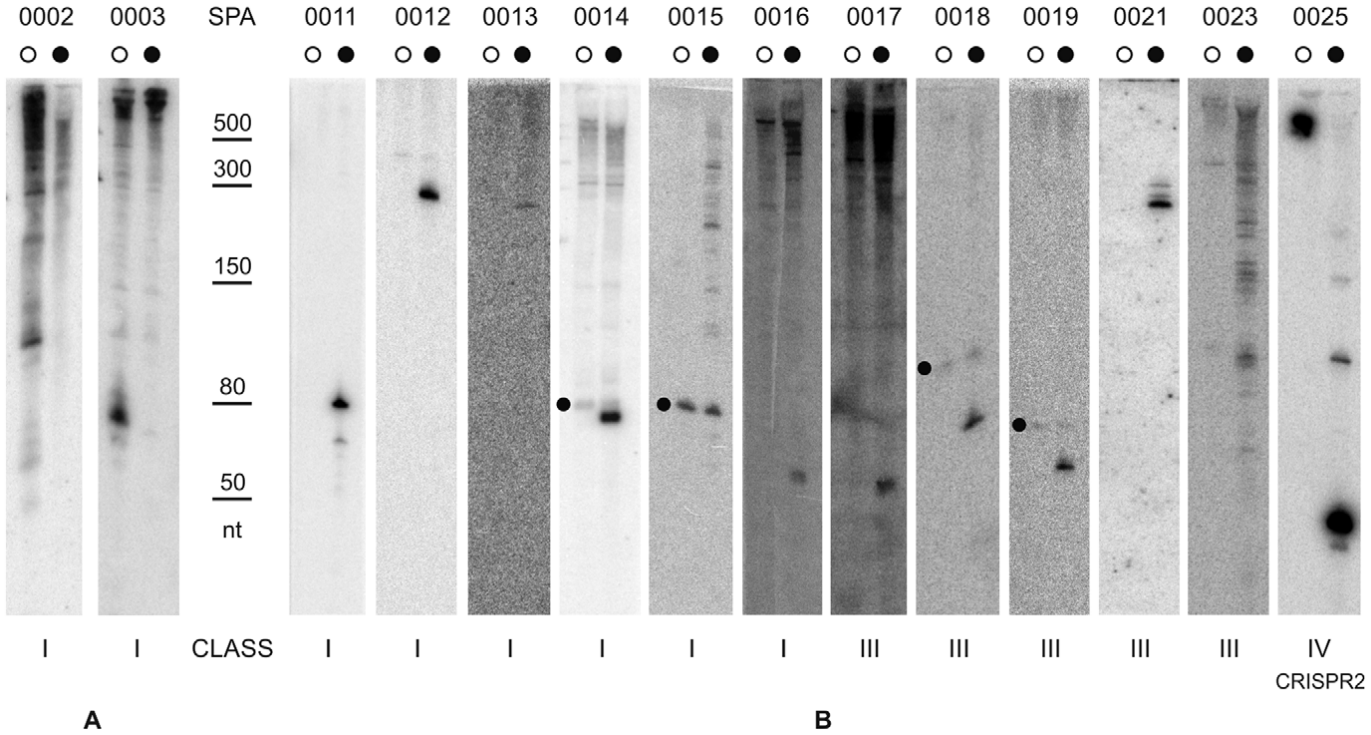
Validation of candidate sRNAs expressed from unique nstSGR in either PAO1 or PA14. A selection of nstSGRs listed in Table S2, unique in either PAO1 or PA14, were inspected by Northern blot for the expression of sRNAs. Total RNA was extracted from both PAO1 (○) and PA14 (•) grown in the same conditions as for sRNA-Seq. Equal amounts of RNA (8 µg) from both strains were blotted and probed with radiolabelled riboprobes (0002 and 0021) or oligos (Table S1) complementary to nstSGR regions with the highest read coverage, as detailed in Materials and Methods. Validated unique sRNAs in PAO1 or PA14 are shown in (A) and (B), respectively. For SPA0014, 0015, 0018, 0019, signals detected in both strains (dots on the left of PAO1 lanes) can be due to aspecific probe hybridization. The ladder of molecular weight markers is shared by (A) and (B). (nt): nucleotides.

**Figure 3:**
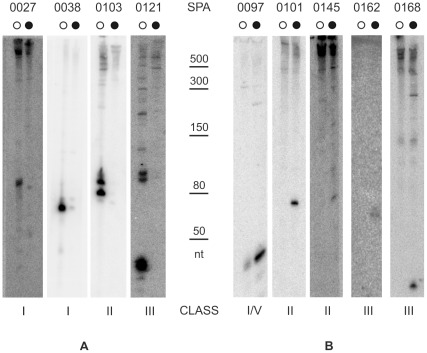
Validation of candidate sRNAs differentially expressed from conserved nstSGR. A selection of conserved nstSGRs listed in Table S2 that were supposed to be differentially expressed between the two strains according to sRNA-Seq data, were inspected by Northern blot. Total RNA was extracted from both PAO1 (○) and PA14 (•) grown in the same conditions as for sRNA-Seq. 8 µg of RNA from both strains were blotted and probed with radiolabelled oligos (Table S1) complementary to nstSGR regions with the highest read coverage. Validated sRNAs which showed higher levels of expression in PAO1 or PA14 are shown in (A) and (B), respectively. The ladder of molecular weight markers is shared by (A) and (B). (nt): nucleotides.

**Figure 4:**
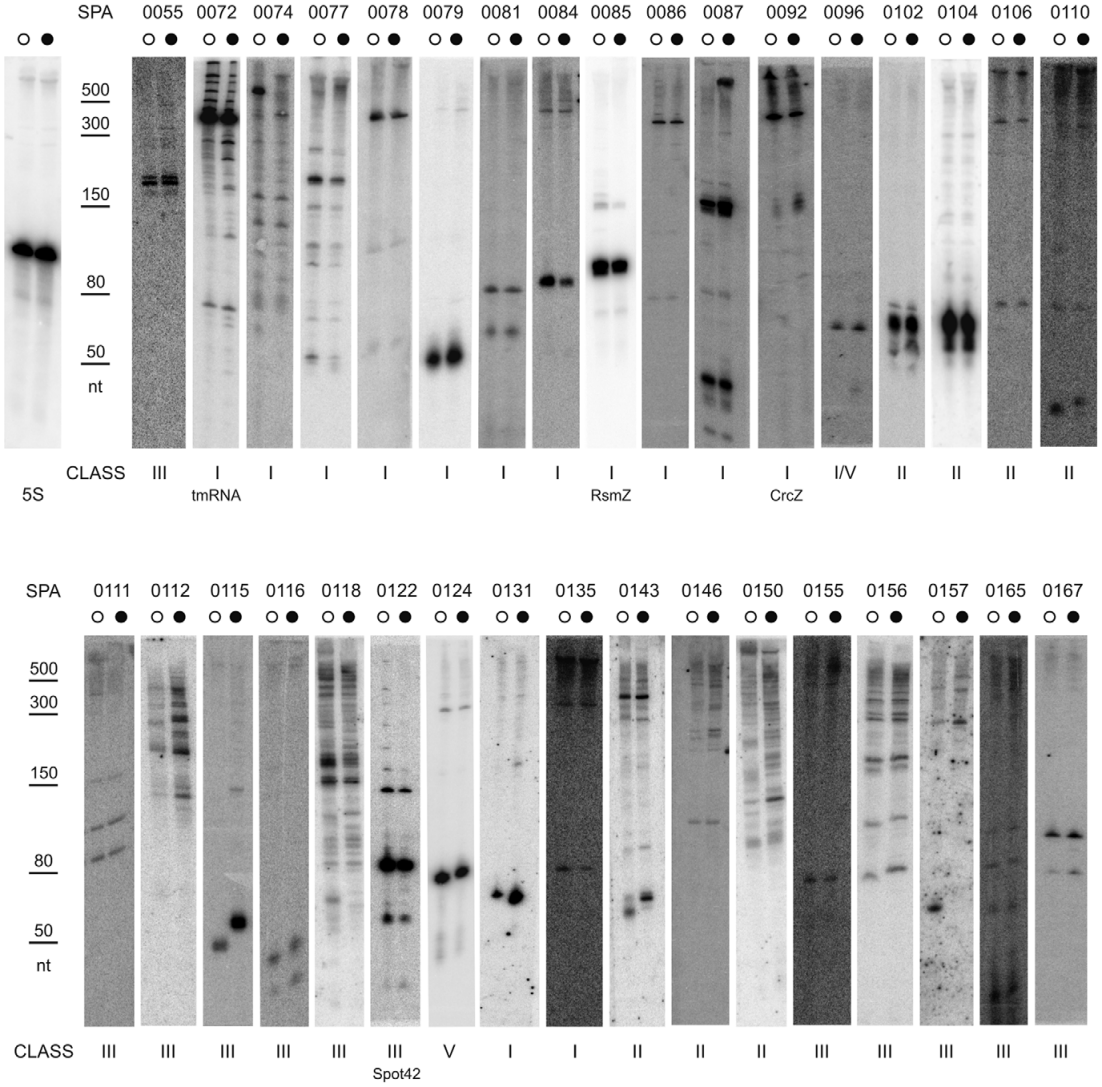
Validation of candidate sRNAs similarly expressed from conserved nstSGR. A selection of conserved nstSGRs listed in Table S2 that were supposed to be similarly expressed between the two strains according to sRNA-Seq data, were inspected by Northern blot. Total RNA was extracted from both PAO1 (○) and PA14 (•) grown in the same conditions as for sRNA-Seq. Equal amounts of RNA (8 µg) from both strains were blotted and probed with radiolabelled oligos or riboprobes (0104, 0112, 0118, 0131, 0143, 0150 and 0157) (Table S1) complementary to nstSGR regions with the highest read coverage. nstSGRs SPA0072, 0085, 0092, and 0122, corresponding to PAO1 *loci* for the known sRNAs tmRNA, RsmZ, CrcZ, and Spot42, respectively, were included in this analysis as positive controls. 5S RNA served as loading control and molecular weight marker. The ladder of molecular weight markers is indicated on the left of each panel. (nt): nucleotides.

cDNA_20–500_ was generated from 1 µg of 5S/tRNA-depleted L1-sRNA_20–500_ using the SMARTer™ PCR cDNA Synthesis Kit (Clontech), which combines RNA reverse transcription with cDNA 3′-end SMART tailing activity, according to manufacturer’s instruction, except that AL1 oligo (Table S1) was used for reaction priming. cDNA was checked by Southern blotting with [^32^P]- labelled oligo SmarterII A (Table S1). RNA template was removed by RNase A digestion. To remove free AL1 oligo, the cDNA preparations were run on preparative dPAGE and AL1-cDNA-SMART ranging from 60 to 540 nt (AL1-cDNA_20–500_-SMART) was electroeluted from gel slices as described above.

**Table 2:** Candidate sRNAs validated by Northern blot.

				PAO1		PA14		
	nstSGR Group^a^	nstSGR name	class	Flanking/Involved *loci* b	strand^d^	Flanking/Involved *loci* b	strand^d^	Notes^e^
Unique	A	SPA0002	I	2326/2327	−			**MRE**
		SPA0003	I	2729/2730	+			**80**
	B	SPA0011	I			30840/*trbI*	+	20
		SPA0012	I			39480/39500	+	**240**
		SPA0013	I			44640/44650	−	40
		SPA0014	I			49480/49500	+	**80**
		SPA0015	I			60120/60130	−	**MRE**
		SPA0016	I			72510/72520	−	**40**
		SPA0017	III			*trbL*	−	**40**
		SPA0018	III			22270	+	50
		SPA0019	III			35720	+	**50**
		SPA0021	III			59370	+	**240**
		SPA0023	III			59840	−	MRE
		SPA0025	IV			33360	−	MRE;CRISPR-2
Conserved	C	SPA0027	I	*toxR*/0708	+	55150/*toxR*		**90**
		SPA0038	I	2754/*eco*	+	*eco*/28486		**70**
		SPA0055	III	0667	+	08540/*tyrZ*		**SR**
	D	SPA0072^c^	I	*ssrA*	−	53560/53570	+	**MRE**; tmRNA
		SPA0074	I	1429/*lasR*	+	*lasR*/45970	−	**MRE**
		SPA0077	I	*bkdR*/*bkdA1*	+	*bkdA1*/*bkdR*	−	50–70
		SPA0078	I	2421/2422	−	33370/33380	+	20–40
		SPA0079	I	2763/2764	−	28350/28360	+	**50**–70
		SPA0081	I	3069/3070	−	*moxR*/24440	+	**90**
		SPA0084	I	3535/3536	+	18620/18630	−	70–50
		SPA0085^c^	I	*rsmZ*	−	*rpoS*/*fdxA*	+	**120**; RsmZ
		SPA0086	I	3919/1920	−	13170/13190	+	70–**430**
		SPA0087	I	4033/*aqpZ*	+	*aqpZ*/11670	−	**170**
		SPA0092^c^	I	*crcZ*	+	*cbrB*/*pcnB*	+	MRE; CrcZ
		SPA0096	I^f^	2751/2752	−	28520	+	50
		SPA0097	I^f^	2771/2770	+	28250	−	**20**
		SPA0101	II	1244	−	48150	+	**80**
		SPA0102	II	*rpsA*	−	*rpsA*	+	MRE
		SPA0103	II	3229	+	22420	−	**100**
		SPA0104	II	*rhlI*	−	*rhlI*	+	**70**
		SPA0106	II	4133	+	*ccoN*	−	**70**
		SPA0110	II	5473	+	72230	+	**30**
		SPA0111	III	*triC*	−	01970	−	70–140
		SPA0112	III	0367	−	04820	−	**MRE**
		SPA0115	III	2759	+	28410	−	70
		SPA0116	III	2769	+	28290	−	**50**
		SPA0118	III	3350	−	*flgA*	+	**MRE**
		SPA0121	III	5480	−	72350	−	**MRE**
		SPA0122	III	5492	−	*engB*	−	30–**90**; Spot42
		SPA0124	V	1414	+	46160	−	**80**
	E	SPA0131	I	*hasAp*/*hasD*		*hasAP*/*hasD*	−	**60**
		SPA0135	I	2559/2560		31430/31440	+	**80**
		SPA0143	II	*pilU*		*pilU*	+	80
		SPA0145	II	*lecB*		*lecB*	−	**90**
		SPA0146	II	3261/3262		21830	+	**120**
		SPA0150	II	*acnA*		*acnA*	+	**140**
		SPA0155	III	*coIII*		*coIII*	−	MRE
		SPA0156	III	*spuA*		*spuA*	−	**MRE**
		SPA0157	III	*ptsP*		*ptsP*	−	120
		SPA0162	III	*alkB1*		*alkB1*	−	**60**
		SPA0165	III	1735		42100	+	**90**
		SPA0167	III	1166/*pcpS*		49330/*pcpS*	−	80
		SPA0168	III	*purC*		*purC*	+	30

a nstSGR group as defined in Table 1: A and B, unique in PAO1 or PA14, respectively; C and E, conserved in both strains but expressed in either PAO1 or PA14, respectively; D, conserved and expressed in both strains.

b Name or number (e.g. 2326 is PA2326, 30840 is PA14_30840) of *loci* in the PAO1 and PA14 genomes either overlapping (class II, 5′-UTR; III, asRNA; and V, sense sRNAs overlapping annotated ORFs) or flanking (class I, sRNA) the nstSGRs.

c Annotated sRNAs, found by sRNA-Seq, used as a positive control in Northern blot validation experiments.

d Upper (+) or lower (−) genomic DNA strand coincident with cDNA reads.

e sRNA size predicted by sRNA-Seq. Single values indicates coherent results between PAO1 and PA14. Otherwise, two values (PAO1-PA14) are reported. Values are indicated in bold whenever confirmed by Northern blotting. MRE: Multiple Read Ends defined by non-overlapping reads scattered within the nstSGR. The name of sRNAs used as validation controls is also indicated.

f Class assignment in PAO1. The corresponding nstSGR in PA14 was assigned to class V.

A cDNA_20–500_-derived amplicon library for 454 pyrosequencing (Roche) was obtained by PCR amplification of AL1-cDNA_20–500_-SMART using Advantage® 2 PCR polymerase (Clontech) with primers (Table S1) tailored for 454-sequencing with Roche Multiplex Identifiers (MID) for “barcoding”. In particular, MID42 (TCGATCACGT) and MID47 (TGTGAGTAGT) were used to tag amplicons from PAO1 and PA14, respectively. To remove free primers, the PCR reactions were run on preparative dPAGE and amplicons ranging from 130 to 610 nt were electroeluted from gel slices as described above. Amplicons quality and length distribution was checked by Southern blot with [^32^P]-labelled SmarterII A oligo and by capillary electrophoresis in Agilent Bioanalyzer DNA 7500 (Figure S2A). To enrich RNA transcripts ranging from 130 to 500 nt, a second sRNA-derived amplicon library (Figure S2B) was generated from 5S/tRNA-depleted L1-sRNA ranging in size from 150 to 520 nt (selected by preparative dPAGE and electroelution from gel slices) as described above.

### Northern and Southern Blot Analyses

The following procedure was used for both Northern and Southern blot analyses. RNA or DNA samples were heated at 95°C for 5 minutes in loading buffer (5 mM EDTA, 0.025% xylene cyanol, 0.025% bromophenol blue dissolved in formamide) and resolved by dPAGE. Nucleic acids were transferred onto Hybond N^+^ nylon membranes (GeHealthcare) using a semi-dry electroblotter apparatus (Fastblot B33, Biometra) set at 25 V, 400 mA for 1 hour. The blots were UV-crosslinked and hybridized with [^32^P]-labelled oligos or riboprobes (Table S1) as described previously [28]. Visualization of radioactive bands was performed by Typhoon™ 8600 variable mode Imager scanner (GE Healthcare BioSciences). All DNA oligonucleotide probes were 5′-end labeled with [γ-^32^P]ATP and T4 polynucleotide kinase. Riboprobes were prepared as described previously [28] by T7 RNA polymerase transcription of DNA templates obtained by PCR using oligos listed in Table S1 and *P. aeruginosa* genomic DNA as template. For each validation of candidate sRNA, the probe was complementary to the cognate genomic region with the highest read coverage.

### 454-pyrosequencing and Data Analysis

Equal amounts of the PAO1 and PA14 amplicon cDNA libraries were combined and submitted to deep-sequencing by a 454 Roche Titanium sequencer using 2/8 of PicoTiterPlate, which should assure at least 140,000 reads. The MID-containing reads were trimmed to eliminate both terminal adaptors, i.e. MIDs, SMART and 454 pyrosequencing primer A-B sequences. Reads were then mapped and clustered throughout the corresponding genome sequence (Genbank accession numbers NC_002516 and NC_008463 for PAO1 and PA14, respectively) as follows. The mapping step was performed using the software SEGEMEHL [29] with default settings but reporting all equal best hits. Mapping positions were considered reliable only if 90% of the read was aligned with ≥90% identity with the genome sequence. Then, the search for read clusters on genome sequences was performed by a sliding window of 200 bp shifted 100 bp at a time along the genome sequence. Significance of clustering of mapped reads was estimated under a null hypothesis of random distribution of reads along the genome using a cumulative Poisson probability. Significant Genomic Regions (SGRs) were defined as consecutive windows where at least one window showed a significant clustering of reads under the Poisson test described above (P≤0.1). SGRs were divided into “structural” (stSGRs, if the genomic annotation reported the keyword “ribosomal” or “tRNA”) and “non-structural” SGRs (nstSGRs, in the other cases). nstSGRs orthology between PAO1 and PA14 strains was determined by reciprocal BLAST. The clustered reads were visualized by GBrowse interface at www.pseudomonas.com database. Sequencing data are accessible at GEO (accession number, GSE36340).

## Results

### Deep-sequencing of the Low Molecular Weight RNA Fraction of *Pseudomonas aeruginosa* PAO1 and PA14 Strains

We aimed at sRNA profiling in the *P. aeruginosa* PAO1 and PA14 strains by sRNA-Seq [26], a massive sequencing approach tailored for unbiased identification of low molecular weight RNA (see Figure S1 for an overview of the procedure). To this end, total RNA was purified from late-exponential cultures of both PAO1 and PA14 strains, respectively, and transcripts ranging from 20 to 500 nt (sRNA_20–500_) were isolated by gel electrophoresis. The 3′ ends of PAO1 and PA14 sRNA_20–500_ were tagged by ligation with linker L1, a mixed ribo-deoxyribo-oligonucleotide with its 3′-end protected by an inverted dT (Table S1), obtaining L1-sRNA_20–500_. sRNA preparations are expected to contain a high proportion of the stable and very abundant tRNAs and 5S rRNA that may interfere with the efficiency of sRNA profiling. We thus selectively degraded the stable RNA component as described previously [26]. Briefly, L1-sRNA_20–500_ was mixed with a pool of DNA oligos (Table S1) complementary to *P. aeruginosa* 5S rRNA and tRNAs, and digested with RNase H. Using the SMARTer™ PCR cDNA Synthesis Kit (Clontech), cDNA_20–500_ was then generated from 5S/tRNA-depleted L1-sRNA_20–500_ by reverse transcription with an oligonucleotide primer complementary to L1 (AL1; Table S1) and the cDNA 3′-end was tailed with a specific sequence (see Materials and Methods for details). An amplicon library for 454 pyrosequencing (Roche) was then generated by PCR amplification of cDNA_20–500_ with modular primers complementary to cDNA ends and carrying sequences tailored for 454 sequencing priming and multiplex identification (MID). The PAO1 and PA14 cDNA_20–500_ amplicons described above were combined in a 1∶1 ratio (amplicon library 1), and submitted to pyrosequencing. This resulted in a raw pool of 101,019 reads (Figure S2A) among which, 0.3% did not show any identifiable linker sequence. The 100,680 linkers-containing reads were examined for MID sequences. 32,156 and 41,514 reads included MID42 (PAO1) and MID47 (PA14) identifiers, respectively, and were at least 17 bases long. After trimming both terminal linker sequences, the reads showed an average length of 34 and 31 nt for PAO1 and PA14, respectively.

As shown in Figure S2A, sRNA molecules longer than 130 nt were poorly represented in this amplicon library. To increase the abundance of longer RNA molecules (corresponding to a read length of about 230 nt in Figure S2A), additional sRNA-derived amplicons were generated for each strain from 150 to 520 nt long RNA fractionated by gel electrophoresis and processed as described above. These PAO1 and PA14 L1-sRNA_150–500_ amplicons were then combined in a 1∶1 ratio, thus producing a second library (amplicon library 2). The pyrosequencing of the latter resulted in a raw pool of 61,490 reads (Figure S2B), among which 59,132 contained identifiable linker sequences. MID analysis showed that 23,608 and 29,107 reads derived from PAO1 and PA14, respectively. Following terminal trimming, the average read length was about 100 nt for PAO1 and 80 nt for PA14.

### Identification of Candidate sRNA *Loci* and Comparative Analysis between PAO1 and PA14 Strains

Under stringent mapping criteria (>90% read coverage aligned at >90% identity to reference genome), 13,438 and 22,691 reads gave at least one satisfactory match with the genome sequences of PAO1 and PA14, respectively (GenBank accession numbers NC_002516 and NC_008463). A non uniform distribution of reads across the genomes was observed. In fact, more than 99% of genomic positions showed zero coverage, while a limited proportion of sites showed high levels of coverage. To map candidate sRNA *loci*, genomic regions showing significant reads clustering, hereafter referred to as significant genomic regions (SGRs), were identified as detailed in Materials and Methods. For each strain, about half of the mapped reads clustered in SGRs overlapping stable RNA genes (i.e. tRNAs, 5S rRNA); these were classified as structural SGRs (stSGRs) and not included in further analysis. Around 90% of the remaining mapped reads fell in other significant clusters (non-structural SGRs, nstSGRs), whereas about 10% were not clustered. As stable RNAs are expected to be much more abundant than other RNAs, the observed 1∶1 ratio between the number of reads mapping in stSGRs over nstSGR reads indicates the high efficiency of tRNAs and 5S rRNA depletion achieved in amplicon library preparation.

As a whole, we defined 128 and 168 nstSGRs in PAO1 and PA14 genomes, respectively (Table 1 and Table S2) mapping within different *loci*: i) genes for housekeeping RNAs (tmRNA, 6S, 4.5S and RNase P RNAs); ii) genes for sRNAs previously identified in PAO1 (14) (Table S3), and iii) both intergenic and intragenic *loci* not previously known to express sRNAs (201).

By reciprocal BLAST, we determined whether the identified *loci* were conserved or not in the two strains. Both unique (in either strain) and conserved *loci* were found. Therefore, the corresponding nstSGRs were classified in 5 groups as shown in Table 1. Group A and B nstSGRs map within *loci* unique to PAO1 and PA14, respectively; groups C, D and E include nstSGRs mapping in conserved *loci*. Group D nstSGRs were found in both strains, whereas group C and E nstSGRs were identified only in PAO1 and PA14, respectively. Thus, the comparative profiling of sRNAs from PAO1 and PA14 suggested the existence of both strain-specific (groups A and B) and conserved candidate sRNA *loci*; the latter, in a number of cases (groups C and E), appeared differentially expressed in the two strains.

Within each group described above, we classified the candidate sRNAs according to functional/structural categories established for regulatory RNAs in bacteria [1] as follows (Figure S3). Class I groups nstSGRs located in intergenic regions (>30 nt from flanking ORFs). *Trans*-encoded sRNAs (sRNA) would belong to this class; class II groups nstSGRs with read clustering spanning 5′-untranslated regions (5′-UTRs) in sense orientation. This class would encompass mRNA riboswitches and sRNAs generated by mRNA transcription attenuation or processing; class III includes nstSGRs with intragenic (<30 nt from flanking ORFs) reads clustering in antisense orientation. *Cis*-encoded antisense sRNAs (asRNAs) would cluster in this class; class IV groups intergenic nstSGRs containing CRISPR-like arrays [30]; finally, nstSGRs with read clustering within ORFs and/or 3′-UTRs in sense orientation belong to class V.

The results of this analysis are summarized in Figure 1 and details of each nstSGRs are listed in Table S2. Since class V nstSGRs may correspond to stable mRNA degradation fragments, whose regulatory role is uncertain, they were excluded from further analysis and not reported in Table S2, with the exception of nstSGRs encompassing small putative ORFs.

Remarkably 19 hits of Table S2 corresponded to members of the panel of about 40 sRNAs previously identified in PAO1 [15] including sRNAs annotated in the *Pseudomonas* genome database_v2_ (www.pseudomonas.com) such as the housekeeping tmRNA, 6S, 4.5S and RNase P RNAs, and sRNAs already characterized such as CrcZ, RsmY, RsmZ, PhrS and AmiL [15] and a putative Spot42 sRNA (SPA0122) which is located in a conserved genomic context in *E. coli*, *Salmonella* and pseudomonads [31] (Table S3). We show here that this panel of known sRNAs previously detected in PAO1 is comparably expressed in PA14. Many of the previously identified sRNAs that escaped our analysis have been reported to be expressed at low level or in response to environmental stimuli (e.g. iron limitation for Prrf1 and 2) [24]. However, in a recent deeper transcriptomic survey of PA14 [32] all known *P. aeruginosa* sRNAs were detected. Therefore, it is possible that in our sRNA-seq approach we missed scarcely expressed sRNAs.

Taken together, the data described above, subtracted from those sRNAs already known in *P. aeruginosa*, represent a panel of 163 novel sRNA candidates.

### Validation by Northern-blot Analysis of sRNAs Expression from nstSGRs

We tested by Northern blotting the expression of a sample of 71 novel candidates covering all groups and classes (Table S2). Our sample for validation was not random, as we gave priority to strain-specific candidates for validation, but disregarding those having features typical of antisense sRNA regulating a transposase genes (see below). Moreover, we favored class I and III candidates, i.e. *trans*-encoded sRNAs and asRNAs, respectively. In particular, we analyzed 29 class I, 12 class II, 29 class III and 1 class V nstSGRs throughout the A-E groups. The previously identified sRNAs RsmZ (SPA0085) [22], CrcZ (SPA0092) [20], Spot42 (SPA0122) [31] and tmRNA (SPA0072) [19] were used as positive controls. Moreover, a class IV nstSGR, corresponding to CRISPR-2 [33] was included in this validation panel.

Out of 71 novel candidates tested, 52 showed signals in Northern blot experiments (Figures 2, 3 and 4); for 19 we could detect only very faint signals, barely above the background, or no signal (data not shown). Thus, the validation rate was above 73%. Among the validated sRNAs (Table 2), 22 belonged to class I (sRNA), 19 to class III (asRNA), 10 to class II (5′-UTR), 1 to class V. The expression of CRISPR-2 (class IV) was validated and a major band, corresponding to processed crRNA [34], was observed.

The majority of sRNAs tested, which were expected to be equally expressed in both strains (group D) (Table S2), showed signals whose intensity in the two strains was consistent with sRNA-Seq data (i.e. the read number of the corresponding nstSGRs). One exception was SPA0101 (Figure 3B) which showed a comparable read number in both strains, but gave a sharp signal corresponding to a ∼70 nt long transcript only in PA14. On the contrary, many group E sRNAs tested (Table S2), whose corresponding nstSGRs displayed expression in PA14 only in sRNA-Seq, showed comparable expression in the two strains in Northern blot analysis. However, all the corresponding nstSGRs of these sRNAs were identified in PA14 by a read number at best slightly above the significance threshold (from 3 to 6). Thus, stochastic fluctuations in amplicon library preparation may have been sufficient to keep the read number below the threshold in PAO1. On the whole, we validated 13 novel unique sRNAs (Figures 2A and B), 30 conserved sRNA with comparable expression in both strains (Figure 4), and 9 conserved sRNAs showing differential expression (Figure 3A and B).

In most cases, we found that transcript size predicted by sRNASeq (Table 2) corresponded to the strongest Northern blotting signal (Figures 2, 3, and 4). The sRNA-Seq reads were scattered within two class I (SPA0015 and SPA0074) and several class II and class III nstSGRs (SPA0023, 0112, 0118, 0150 and 0156). Accordingly, these nstSGRs showed complex patterns with multiple signals of comparable intensity. Degradation of unstable primary transcripts by cellular nucleases may explain these results. However, the presence of scattered reads within an nstSGR was observed also for other sRNAs such as CRISPR2, tmRNA, CrcZ and SPA102, for which a major signal was clearly visible by Northern hybridization. Moreover, in some cases (i.e. SPA0011, 0013, 0018, 0157, 0167, 0168), the regions covered by the reads were smaller than the observed transcripts. As samples preparation for sRNA-Seq included an RNase H digestion step, unspecific RNA degradation by this enzyme may account for these results.

## Discussion

### Increasing Complexity of *P. aeruginosa* RNA World by sRNA-Seq

In this work we have performed a parallel sRNAs search in *P. aeruginosa* by sRNA-Seq, a powerful unbiased method that allows the analysis by deep sequencing of the whole small transcriptome (*i.e.* both primary and processed transcripts) [26]. Unlike previous surveys performed in PAO1, our search for sRNA *loci* was not biased by *a priori* assumptions about sRNA-based regulation mechanisms, such as binding by Hfq [35], whose role in sRNA-mediated regulation system is not clearly established in *P. aeruginosa*, or genetic features putatively associated with sRNA-coding *loci* (*e.g.* mapping within intergenic regions with predicted promoters and terminators), which were employed in previous bioinformatics-based analyses [17]–[19]. The first goal of our analysis was to expand the *P. aeruginosa* sRNA panel resulting from previous surveys in terms of both amplitude and sRNA typologies (potential antisense RNAs for example were completely disregarded by previous analyses) [15], [17]–[19]. Moreover, we did not overlook putative “bifunctional” sRNAs, such as short transcripts encompassing 5′-UTRs or encoding small peptides [3], [36].

Our approach resulted in the definition of 163 *loci* expressing new candidate sRNAs. We found a comparable number of class I (sRNA) and III (asRNA) sRNAs, which altogether accounted for more than 75% of our sRNAs panel (Figure 1). In addition, several (34/181) class II sRNAs (mapping within 5′-UTR) were found. These short transcripts, also identified in previous genome-wide searches for sRNAs [37], [38], can be generated by premature transcription termination, or 5′-UTR processing as by-products of post-transcriptional gene regulation or mRNA degradation. However, in some cases they can also act as *trans*-encoded sRNAs. In fact, it has recently been reported that two S-adenosylmethionine riboswitches of *Listeria monocytogenes*, SreA and SreB, can base pair with the mRNA of *prfA*, the master regulator of *Listeria* virulence, and repress its expression [39].

Finally, both the annotated CRISPR of PA14 [33] and ten class V *loci* for potential peptide-coding sRNAs were detected (Figure 1). These latter sRNAs may have the dual *status* of short mRNAs (encoding low molecular weight proteins) and *trans*-acting sRNAs, as it has been established for the *E. coli* SgrS and the *Staphylococcus aureus* RNAIII sRNAs [40]–[42].

We assayed the expression of a large sample of candidate sRNAs by Northern blotting. Remarkably, the expression of many ntsSGRs defined by a read number only slightly above the significance threshold (e.g. SPA0102, 0110, 0112 and 0156) could be demonstrated by Northern blotting. Furthermore, the majority of validated sRNAs showed expression levels in Northern assays that were consistent with sRNA-Seq analysis. Thus sRNA-Seq not only appears a sensitive approach to sRNA identification but could also represent a reliable method for estimating their expression levels in comparative analyses.

On the whole, we could validate the expression of 52 novel sRNAs, more than doubling the number of *P. aeruginosa* sRNAs annotated so far. Interestingly, several validated 5′-UTR nstSGRs (e.g. SPA0101-0104) showed one or two sharp signals in Northern blotting experiments (Figs. 2, 3, and 4) corresponding to discrete RNA species and may thus be good candidates for *trans*-acting sRNAs, as mentioned above. Overall, our data significantly increase the complexity of sRNA complement in *P. aeruginosa* and suggest that RNA-mediated regulation in this organism may be as common and multifaceted as it is in other bacteria [1], [3].

### sRNA-mediated Regulation May Contribute to *Pseudomonas* Strain-specific Phenotypic Traits

Another purpose of our work was to get hints on sRNA-mediated regulatory mechanisms possibly involved in strain-specific phenotypic traits such as pathogenicity and virulence. To this aim, we performed a comparative analysis of PAO1 and PA14 strains that, although sharing the same host range, differ in virulence, being PA14 considerably more virulent in several model organisms [43].

26 nstSGRs identified by sRNA-Seq consisted of unique *loci* in either PAO1 or PA14 (groups A and B, respectively; Table S2). In PA14, these *loci* mostly mapped within regions of genome plasticity (RGPs, defined as polymorphic strain-specific segments encompassing at least 4 contiguous ORFs) [9], with SPA0016 representing the only exception (Table S2). As for the 9 nstSGRs unique to PAO1, 2 mapped in RGPs (SPA0001, which corresponded to the already known PhrD sRNA [15], [19] and SPA0003. Remarkably, 6 overlapped in antisense orientation the 5′-UTRs of a gene encoding a putative transposase of the IS*116*/IS*110*/IS*902* family (SPA0004-8 and SPA0066). This gene is identically repeated six times in PAO1 genome and sRNA-Seq reads were randomly distributed by the mapping software among the six *loci*. Transposase translation regulation by antisense RNAs has been extensively studied in the IS*10* system, where a short RNA (RNA-OUT), which is transcribed in antisense orientation from the 5′-end of the transposase locus, interacts with transposase mRNA to hinder ribosome binding site [44], [45]. We did not check by Northern blotting the expression of this PAO1 putative transposase antisense RNA; however, the high overall sRNA-Seq read number (899; Table S2) suggests that it can be actively transcribed and thus may play a role in transposase regulation. Another sRNA antisense to a transposase gene could be expressed by the SPA0022 *locus*, which maps within the PAPI-1 pathogenicity island unique to PA14 and encodes a polypeptide belonging to IS*66* OrfC family [46]. However, in this case only few reads were detected by sRNA-Seq that mapped within the 3′-end region of the transposase.

We validated the expression of 13 novel strain-specific sRNAs by Northern blotting, 2 unique to PAO1 and 11 to PA14. Interestingly, three PA14 novel sRNAs, SPA0015, SPA0021 and SPA0023, fall within the pathogenicity island PAPI-1. SPA0015 *locus* maps in the intergenic region between genes *RL003* (PA14_60130), encoding a homolog of DNA relaxase [47], and *RL004* (PA14_60120; *dcd2*), encoding a putative deoxycytidine deaminase. *RL003* mutants showed virulence-attenuated phenotype [48], and reduced efficiency in PAPI-1 horizontal transfer [47]. SPA0021 and SPA0023 asRNAs are *cis*-encoded antisense to *RL076* (PA14_59370) and *RL033* (PA14_59840) genes, respectively, both encoding hypothetical proteins. An insertion mutation in *RL076* reduced the efficiency of PAPI-1 horizontal transfer [47], whereas an *RL033* mutant showed attenuated virulence [48]. It will be interesting to assess whether sRNA-mediated regulation at these *loci* may be involved in PA14 virulence.

Most nstSGRs (155/181; Table S2) mapped in conserved *loci* and were identified by sRNA-Seq in both strains (group D) or in either one (groups C and E). At the pangenome level, these conserved *loci* mostly belong to the core genome, which constitutes approximately 90% of the total genome and is highly conserved in all strains analyzed so far [9], [10]. Only three conserved *loci*, SPA0097, SPA0169 and SPA182 belong to the accessory genome and mapped within RGPs (Table S2). Out of the 52 novel validated sRNAs *loci*, 30 belong to the core genome and exhibited comparable expression in PAO1 and PA14 (Figure 4). On the contrary, 8 sRNAs belonging to the core genome and 1 to RPG43 showed differential expression between the two strains, being more highly expressed either in PAO1 (Figure 3A) or in PA14 (Figure 3B). It has been pointed out that for sRNAs with multiple targets, a hierarchy in target binding due to sRNA-mRNA interaction strength may exist [49]. Thus, it is possible that differential expression of a sRNA may result not only in quantitative differences in the strength of target mRNA(s) regulation, but also ultimately change the number of mRNA species targeted by the same sRNA. It will be interesting to assess whether this is indeed the case for *P. aeruginosa* differentially expressed sRNAs.

## Supporting Information

Figure S1 Steps of the comparative analysis of the small transcriptome of PAO1 and PA14 strains. From RNA extraction to sRNAs verification, the sequence of steps followed for the comparative analysis of the small transcriptome of the strains PAO1 and PA14 is depicted as a flow chart. The whole approach was performed in three phases: (A) RNA preparation and 454 pyrosequencing; (B) Deep-sequencing data analysis; (C) Comparative PAO1 *vs* PA14 analysis.
(TIF)

Figure S2 Read length distribution of amplicon libraries. 454 pyrosequencing results in terms of length distribution of untrimmed reads are shown for amplicon library 1 (A) and 2 (B). Terminal adaptors of amplicons for pyrosequencing are altogether 100 nt long. Note the enrichment in (B) of reads with actual length longer than 130 nt, which are scarcely represented in (A).
(TIF)

Figure S3 Criteria for categorization of nstSGRs into classes. nstSGRs are represented by thick black arrows. By way of example, the upper nstSGR bears on top the read cluster by which nstSGR has been defined. Grey tip-ended segments represent annotated ORFs located farther than 30 nt from the nearest end of read cluster. Orange tip-ended segments represent annotated ORFs located within 30 nt from or overlapping with (ol) at least one end of the read cluster. Class I (intergenic), II (5′-UTRs), III (antisense) and V (intragenic) nstSGRs differ for their relative positioning to annotated ORFs. Class IV nstSGRs corresponding to CRISPR-like array are not depicted in this figure.
(TIF)

Table S1 Oligonucleotides.
(PDF)

Table S2 Compilation of nstSGRs identified by parallel sRNA-Seq approach in the *P. aeruginosa* strains PAO1 and PA14.
(PDF)

Table S3 Previously identified *P. aeruginosa* sRNAs found in this work.
(PDF)
