# Supplementary figures and images for: Comparative Profiling of Pseudomonas aeruginosa Strains Reveals Differential Expression of Novel Unique and Conserved Small RNAs
Source: PLoS One. 2012 May 10;7(5):e36553. doi: 10.1371/journal.pone.0036553 (PMC3349714; doi:10.1371/journal.pone.0036553)

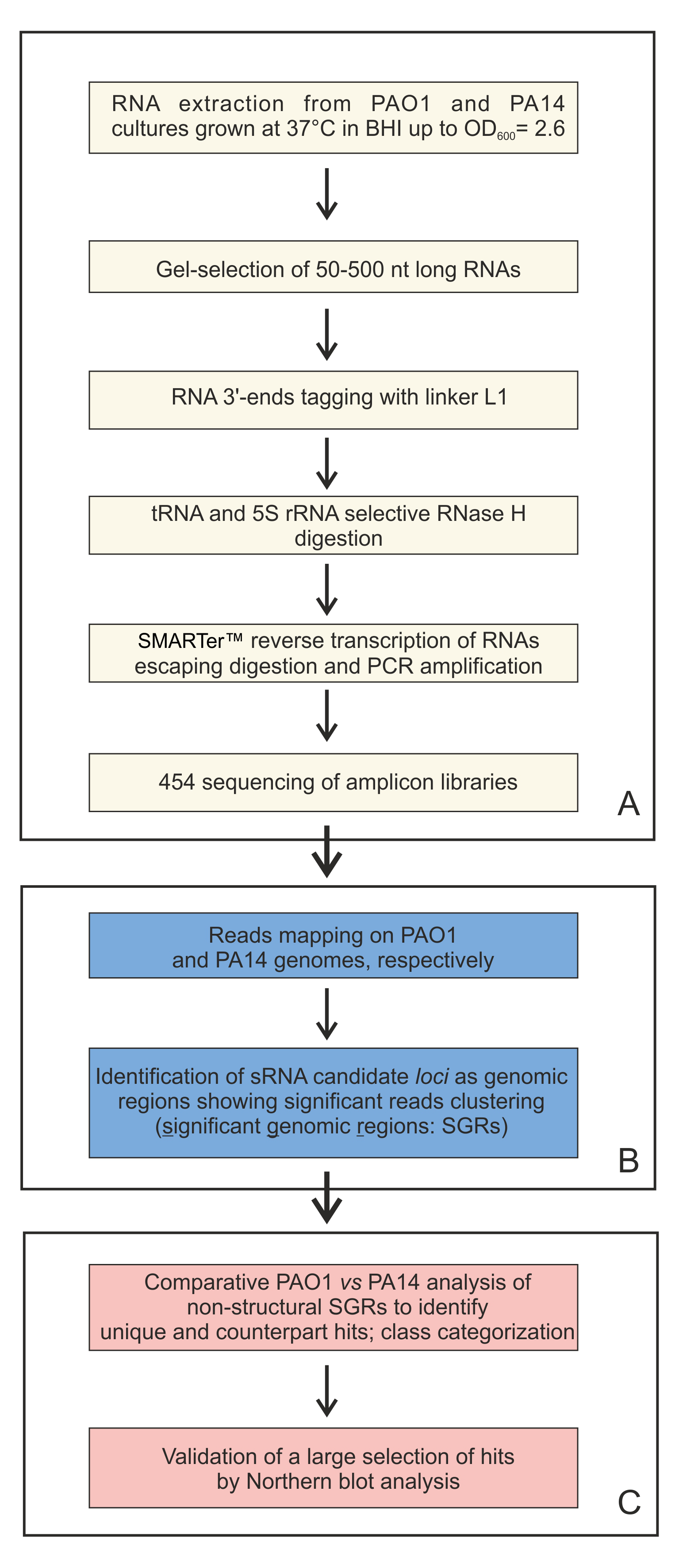

Supplement: Figure S1 — Steps of the comparative analysis of the small transcriptome of PAO1 and PA14 strains. From RNA extraction to sRNAs verification, the sequence of steps followed for the comparative analysis of the small transcriptome of the strains PAO1 and PA14 is depicted as a flow chart. The whole approach was performed in three phases: (A) RNA preparation and 454 pyrosequencing; (B) Deep-sequencing data analysis; (C) Comparative PAO1 vs PA14 analysis. (TIF) [file pone.0036553.s001.tif]

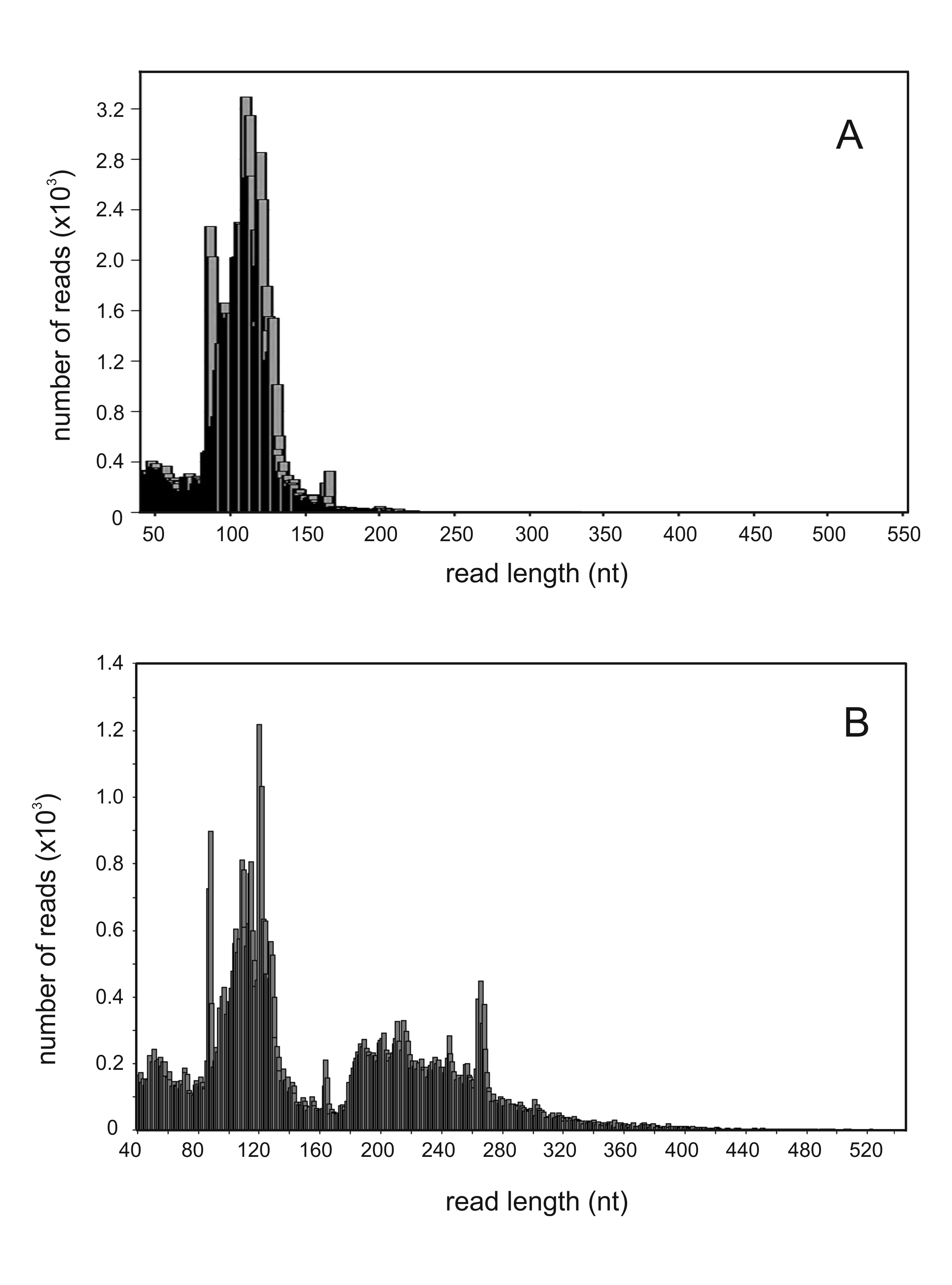

Supplement: Figure S2 — Read length distribution of amplicon libraries. 454 pyrosequencing results in terms of length distribution of untrimmed reads are shown for amplicon library 1 (A) and 2 (B). Terminal adaptors of amplicons for pyrosequencing are altogether 100 nt long. Note the enrichment in (B) of reads with actual length longer than 130 nt, which are scarcely represented in (A). (TIF) [file pone.0036553.s002.tif]

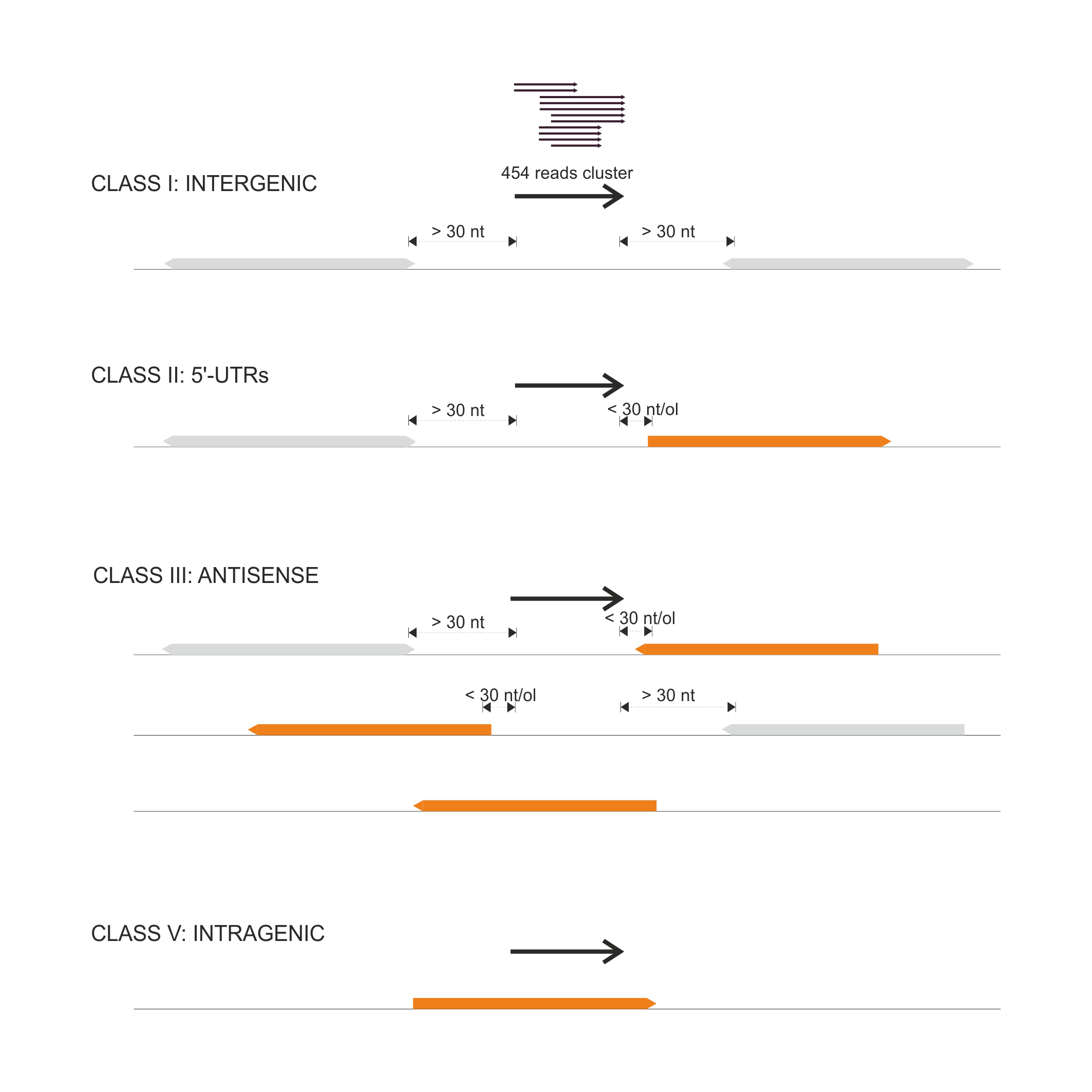

Supplement: Figure S3 — Criteria for categorization of nstSGRs into classes. nstSGRs are represented by thick black arrows. By way of example, the upper nstSGR bears on top the read cluster by which nstSGR has been defined. Grey tip-ended segments represent annotated ORFs located farther than 30 nt from the nearest end of read cluster. Orange tip-ended segments represent annotated ORFs located within 30 nt from or overlapping with (ol) at least one end of the read cluster. Class I (intergenic), II (5′-UTRs), III (antisense) and V (intragenic) nstSGRs differ for their relative positioning to annotated ORFs. Class IV nstSGRs corresponding to CRISPR-like array are not depicted in this figure. (TIF) [file pone.0036553.s003.tif]
