# Supplementary material for: Comparative Profiling of Pseudomonas aeruginosa Strains Reveals Differential Expression of Novel Unique and Conserved Small RNAs
Source: PLoS One. 2012 May 10;7(5):e36553. doi: 10.1371/journal.pone.0036553 (PMC3349714; doi:10.1371/journal.pone.0036553)
Supplement: Table S1 — Oligonucleotides. (PDF) [file pone.0036553.s004.pdf]

Table S1. Oligonucleotides.

**Oligo Name**	**Sequence (5' → 3')**
sRNA-Seq amplicon libraries generation[a]	
L1[b]	5'-P-rGrCrUAGTTACTCACACTAGTGTCC/invdT
AL1	GGACACTAGTGTGAGTAACTAGC
SMARTerII A	AAGCAGTGGTATCAACGCAGAGTA
454forAal1MID42	CGTATCGCCTCCCTCGCGCCATCAGTCGATCACGTCTAGT GTGAGTAACTAGC
454revBsmarterIIMID42	CTATGCGCCTTGCCAGCCCGCTCAGTCGATCACGTGTGGT ATCAACGCAGAGTA
454forAal1MID47	CGTATCGCCTCCCTCGCGCCATCAGTGTGAGTAGTCTAGT GTGAGTAACTAGC
454revBsmarterIIMID47	CTATGCGCCTTGCCAGCCCGCTCAGTGTGAGTAGTGTGGT ATCAACGCAGAGTA
5S rRNA and tRNA depletion	
PA5SRNA01	GAGCTTGACGATGACCTACTCTCACATG
PA5SRNA02	GGAGACCCCACACTACCATCGGCGATG
PAtRNA03	TGGCGGAGAGGGGGGATTCGAACCCCCGA
PAtRNA05	TGGCGCACTCAGGAGGATTCGAACCTCCGA
PAtRNA06	TGGTGGGTCGTGTAGGATTCGAACCTACGA
PAtRNA07	TGGCGGAGAGATAGGGATTTGAACCCTAGG
PAtRNA08	TGGCGCACCCGGCAGGACTCGAACCTGCGA
PAtRNA09	TGGGGTGGACGATGGGAATCGAACCCACGA
PAtRNA10	TGGTGCGGACGGAGAGACTCGAACTCTCAC
PAtRNA12	TGGCGGAGCGGACGGGACTCGAACCCGCGA
PAtRNA13	TGGCGGTGAGGGAGGGATTCGAACCCTCGA
PAtRNA14	TGGTCGGAGCGACTGGATTCGAACCAGCGA
PAtRNA16	TGGTGCCTCGGGAGAGACTCGAACTCTCAC
PAtRNA17	TGGACGTTCGAGCGGGATTCGAACCCGCGA
PAtRNA18	TGGCAGGGGCGGCTGGATTCGAACCAACGC
PAtRNA19	TGGTGGCTACACCGGGACTTGAACCTGGGA
PAtRNA21	TGGTGCCCAGGAGAAGACTCGAACTTCCAC
PAtRNA22	TGGTGCCCAGGGACGGAATCGAACCGCCGA
PAtRNA23	TGGTGCCGGCACCAGGAGTCGAACCCGGGA
PAtRNA26	GGAAGGCAGTGGGAGTCGAACCCACCC
PAtRNA27	TGGTACCGAGGAGGGGACTCGAACCCCTA
PAtRNA28	TGGTTGCGGGGGCTGGATTTGAACCAACGA
PAtRNA31	TGGCTCCGCGACCTGGACTCGAACCAGGGA
PAtRNA32	TGGTCGGGGTAGAGAGATTCGAACTCCCGA
PAtRNA34	TGGTGCCGGATAGAGGAATCGAACCCCCGA
PAtRNA35	TGGTGGGTCTGGGCAGATTCGAACTGCCGA
PAtRNA36	TGGTGGAGCCAAGGAGGATCGAACTCCTGA
PAtRNA37	TGGTGGAGGGAGAAGGATTCGAACCTTCGA
PAtRNA38	TGGAGCGGGTAGCGGGAATCGAACCCGCA
PAtRNA39	TGGAGCTCATGAGCGGATTTGAACCGCTGA
PAtRNA40	TGGCAGGCCAGGAGGGAATCGAACCCCCAA

Oligo Name	Sequence (5' → 3')
PAtRNA41	TGGCGTCCCGGAGAGGGGTCGAACCTCCAA
PAtRNA46	TGGTGGAGCTAGACGGGATCGAACCGTCGA
PAtRNA48	TGGTGGGTGATGACGGGATCGAACCGCCGA
PAtRNA50	TGGCGCAGCGGACGGGACTCGAACCCGCGA
PAtRNA51	TGGCGTCCCCTAGGGGACTCGAACCCCTGT
PAtRNA54	TGGTAGGCACGATTGGATTCGAACCAACGA
PAtRNA55	TGGTCGGGACGGAGTGATTCGAACACTCGA
PAtRNA56	TGGCGGAGGCGGTGAGATTCGAACTCACGG
PAtRNA57	TGGAGCGGGAAACGAGACTCGAACTCGCGA
PAtRNA58	TGGAGGCTGAGGTCGGAATCGAACCGGCGT
PAtRNA59	TGGTGCCCGGAGCCGGGGTCGAACCGGCAC
PAtRNA60	TGGTTGCGGGAGCTGGATTTGAACCAACGA
PAtRNA61	TGGTGGAGCCGGGGGGATTTGAACCCCCGT
PAtRNA62	TGGAGCGGGCGAAGGGAATCGAACCCTCG
PAtRNA63	TGGTGGGCCCACACGGACTCGAACCGTGGA
PAtRNA64	TGGCGCATCCGGCGGGATTCGAACCCACGA
Northern blotting validation probes[c]	
5S	CGCCGATGGTAGTGTGGGGTC
SPA0003	GGCGCTTGAACACCGCTC
SPA0010	ACTGGAACGCCGTCAGGT
SPA0011	TGCCCGAGGCCGGAATCG
SPA0012	CAATTACCGGCGCGGTAGG
SPA0013	GAAGAAGCCCGCAGTAGCG
SPA0014	GGGTGCCGGGAGTTAAGA
SPA0015	TGCGATGCAATTACGCAGTTG
SPA0016	GGTAAGGCCGAGCTGACA
SPA0017	GCGCGTCGGCCGGCGT
SPA0018	AAAATCGGCGAAGCCACTAAAGCACT
SPA0019	CTGGCTAAGGGAGCGGCA
SPA0023	GCCCTCATCAACTCTGCCAAAGAC
SPA0025	TACACGGCAGTGAACACCGCGC
SPA0027	CCGAGCCGATCCCCTACC
SPA0033	AGCCGCAGGTGCAGGAAC
SPA0038	AAAGACCATCTGCGGGGG
SPA0054	CTTGCCGGAGCGGCAAGG
SPA0055	GCAATCCGATCAGAAAGCGC
SPA0056	GGCTGGCGGAGAGCGCTATC
SPA0061	CGGCAACATCGTCGAGAGCGAC
SPA0070	GCCCGGGAGCTCAGCGGA
SPA0071	GTAACCCTGATGGTAGAGCCC
SPA0072	GGTGGAGCCGGGGGGATT
SPA0074	CGCCCGTGCCCGACGAC
SPA0077	CGGTCGTACTGGGTGACG
SPA0078	ACGCGGGCCGCAGGTGGT

Oligo Name	Sequence (5' → 3')
SPA0079	ACCCCTCCATGCCCGTCG
SPA0080	GGGAAAGCCCCGGAGGG
SPA0081	AGCAGCCCACCGACCCAG
SPA0084	AGGTGCGGATCTCCGGGG
SPA0085	ATCGTCCTGATGAATCGCCTCCCT
SPA0086	CTGACAGCAGAGGTGAGG
SPA0087	ACATCCCTGTGTCGGAGCA
SPA0088	CCAGCGAGCGCGACATGG
SPA0092	GAAGCTCCCCCCCAAGTAG
SPA0096	GGGACCACAGCGGCAACT
SPA0097	AATCCCGGCCGCGTGGAG
SPA0100	CCAGCCAGGCCGGCGAG
SPA0101	CAGGATCGCGCAGGGTTG
SPA0102	ACGGCGCATTCGTGGACC
SPA0103	GTACCGAGCACTGGCATCC
SPA0106	AGCCGGCGACCGCCGTC
SPA0110	CCCCGCGGGAGTTCGTC
SPA0111	CGCCGCCGTAGCAGAGTC
SPA0113	TGGAACAGGCCGAGCGTGCC
SPA0114	CTGAACTTGCAGCTCATCACTGGG
SPA0115	GAAGAGGGAAGAGCTCCGGCC
SPA0116	AAATCCCGACCGCGGGG
SPA0117	GCGGAGCCCTTGGGCTTG
SPA0119	TCTGCGTAACGTCCAGCTGCAG
SPA0121	ACGCAAGGCCGTCCGGCAC
SPA0122	CCCCGAGCTTCGTATGGG
SPA0124	GGAAAGAGTAGACCGGCGT
SPA0129	CGAGCATGCAACGCGGGA
SPA0135	GCCAAGGCGCAAGCCTGAA
SPA0145	GCCGTCTGGCGCGCGGTA
SPA0146	GGATAACCTTGTGAACAGCCC
SPA0147	ACACACGACTCTCACGGTCG
SPA0155	GGCGCCCACGTGACCCT
SPA0156	GCTGATCATTCCTGCGCTGGG
SPA0160	CGGATGAGGCTTCCATGCTG
SPA0162	GGCGAGGACGGGCGCTA
SPA0163	TTGCTGGTGACCCTGCGC
SPA0165	TGACCTGGTGGCTGCCTG
SPA0167	AAGGTCGAGACAGGACAGTATC
SPA0168	GTTCGGCCTGTTCCATGGCCAG
SPA0174	AGCGCTGAGGCTTGCGAC
DNA templates amplification for riboprobe synthesis[c,d]	
SPA0002F	CCCTACGGGTTACGAGGAGCTC
SPA0002R	**CTAATACGACTCACTATAGGG**GGATCAACACATTCG

**Oligo Name**	**Sequence (5' → 3')**
SPA0021F	CTCAAGAGGTTCGGTGTTTCCTCGATTTAAGGG
SPA0021R	**CTAATACGACTCACTATAGGG**CAGGAGCATCAGGCT
SPA0104F	ATCTGGCAGGTTGCCTGCCGTTCATCCTC
SPA0104R	**CTAATACGACTCACTATAGGG**AGTCCCCGTGTCGTG
SPA0112F	GATCGCGGGTCTTGATGCGTGGTGC
SPA0112R	**CTAATACGACTCACTATAGGG**CCGCTTTGTGTACCT
SPA0118F	GCGCCCGTCGAGTCCGCTATTCTGC
SPA0118R	**CTAATACGACTCACTATAGGG**GGCTCGGCTACCTGT
SPA0131F	GGACATTGCGGCAGTCTCC
SPA0131R	**CTAATACGACTCACTATAGGG**TGGCGCTCCTCTCCGGC
SPA0143F	TAGGCGCATTCCTACCTATCCTTGC
SPA0143R	**CTAATACGACTCACTATAGGG**CCTTTTCCACCATCA
SPA0150F	GATCGGGCCACCGCGCATTACC
SPA0150R	**CTAATACGACTCACTATAGGG**AAAGCAGGCATTTCT
SPA0157F	CATGGCGCGGGTCTGGACCAGGAAG
SPA0157R	**CTAATACGACTCACTATAGGG**CGGCGACAAGGCGCT

[a] The MID identifier sequence is underlined.
[b] Ribo-deoxyribo-oligonucleotide with an inverted dT at the 3'-end.
[c] The oligo name corresponds to the cognate nstSGR.
[d] The T7 RNA polymerase promoter sequence is reported in bold.
