# Supplementary material for: Comparative Profiling of Pseudomonas aeruginosa Strains Reveals Differential Expression of Novel Unique and Conserved Small RNAs
Source: PLoS One. 2012 May 10;7(5):e36553. doi: 10.1371/journal.pone.0036553 (PMC3349714; doi:10.1371/journal.pone.0036553)
Supplement: Table S2 — Compilation of nstSGRs identified by parallel sRNA-Seq approach in the P. aeruginosa strains PAO1 and PA14. (PDF) [file pone.0036553.s005.pdf]

Table S2. Compilation of nstSGRs identified by parallel sRNA-Seq approach in the *P. aeruginosa* strains PAO1 and PA14.

		PAO1				PA14				
**nstSGR name[e]**	**class**	**Genomic coordinates (left-right)**	**Flanking/ Involved *loci*[a]**	**# reads[b]**	**strand[c]**	**Genomic coordinates (left-right)**	**Flanking/ Involved *loci*[a]**	**# reads[b]**	**strand[c]**	**Notes[d]**
Group A										
SPA0001	I	785400-785600	*phrD*	95	+					PhrD; RGP5
**SPA0002**	I	2568400-2569000	PA2326/PA2327	6	-					
**SPA0003**	I	3087500-3087800	PA2729/PA2730	36	+					RGP28
SPA0004	III	501100-501300	PA0445	150	+					
SPA0005	III	2556800-2557000	PA2319	149	-					
SPA0006	III	3044700-3044900	PA2690	151	+					
SPA0007	III	3843200-3843400	PA3434	150	+					
SPA0008	III	5383800-5384000	PA4797	150	+					
SPA0066	III	4473500-4473700	PA3993	149	-					
Group B										
SPA0009	I					278200-278400	PA14_03160/PA14_03170	7	+	RGP2
**SPA0010**	I					1924300-1924500	PA14_22090/PA14_22100	6	-	RGP33
**SPA0011**	I					2677700-2678200	PA14_30840/*trbI*	11	+	RGP26
**SPA0012**	I					3515400-3515700	PA14_39480/PA14_39500	152	+	RGP52
**SPA0013**	I					3974900-3975200	PA14_44640/PA14_44650	5	-	RGP16
**SPA0014**	I					4401600-4401700	PA14_49480/PA14_49500	8	+	RGP47
**SPA0015**	I					5355400-5355700	PA14_60120/PA14_60130	10	-	RGP41; PAPI-1
**SPA0016**	I					6460900-6461400	PA14_72510/PA14_72520	5	-	
**SPA0017**	III					1315700-1316500	*trbL*	57	-	RGP36
**SPA0018**	III					1940100-1940300	PA14_22270	7	+	RGP33
**SPA0019**	III					3176100-3176300	PA14_35720	7	+	RGP23
SPA0020	III					4134300-4134700	PA14_46460	7	+	RGP14
**SPA0021**	III					5288100-5288500	PA14_59370	9	+	RGP41; PAPI-1
SPA0022	III					5303200-5303500	PA14_59580	5	-	RGP41; PAPI-1
**SPA0023**	III					5330700-5330900	PA14_59840	5	-	RGP41; PAPI-1
SPA0024	IV					2926400-2927500	PA14_33290/PA14_33300	3	+	RGP24; CRISPR-1
**SPA0025**	IV					2935700-2937400	PA14_33360	23	-	RGP24; CRISPR-2

		PAO1				PA14				
nstSGR name[e]	class	Genomic coordinates (left-right)	Flanking/ Involved *loci*[a]	# reads[b]	strand[c]	Genomic coordinates (left-right)	Flanking/ Involved *loci*[a]	# reads[b]	strand[c]	Notes[d]
Group C										
SPA0026	I	580100-580200	*nirS*/*nirQ*	3	-	590077-590177	*nirS*/*nirQ*			
**SPA0027**	I	781000-781300	*toxR*/PA0708	11	+	4899097-4899397	PA14_55150/*toxR*			
SPA0028	I	912700-912900	PA0836.1	5	-	4738703-4738903	PA53450			P5
SPA0029	I	1028100-1028200	PA0937/PA0938	6	+	4623357-4623457	PA14_52130/*yaiL*			
SPA0030	I	1097000-1097300	PA1013.1/PA1014	5	-	4552631-4552931	PA14_51220/PA14_51230			
SPA0031	I	1135200-1135400	PA1047/PA1048	4	+	4514530-4514730	PA14_50810/PA14_50820			
SPA0032	I	1251200-1251300	*nrdB*/PA1156	5	-	4397712-4397812	*nrdA*/*nrdB*			
**SPA0033**	I	1474300-1474400	PA1361/PA1362	4	-	4160214-4160311	PA14_46670/*norM*			
SPA0034	I	2031800-2031900	PA1869/PA1870	4	+	3601268-3601369	PA14_40300/PA14_40310			
SPA0035	I	2330400-2330600	*ada*/PA2119	5	-	3312492-3312692	PA14_37170 /*ada*			
SPA0036	I	2558300-2558900	PA2319/*gntR*	2	-	3078836-3079153	*gntR*/PA14_34670			
SPA0037	I	2614800-2614900	PA2364/PA2365	7	+	3029702-3029801	PA14_34070/PA14_34080			
**SPA0038**	I	3116400-3116600	PA2754/*eco*	11	+	2457373-2457573	*eco*/PA14_28486			
SPA0039	I	3261300-3261400	PA2906/*cobl*	4	-	2307957-2308057	*cobL*/PA14_26485			
SPA0040	I	3489900-3490100	*purF*/PA3109	4	-	2078190-2078390	*cvpA*/*purF*			
SPA0041	I	4094900-4095000	*rpsB*/*map*	3	-	1463019-1463119	*map*/*rpsB*			
SPA0042	I	4780700-4780900	PA4270.1	4	-	745920-746120	*rplL*/*rpoB*			P26
SPA0043	I	5121600-5121800	PA4573/PA4574	4	-	5394065-5394265	PA14_60520/PA14_60530			
SPA0044	I	5325500-5325700	*pnp*/*rps0*	8	-	5597429-5597629	*rps0*/*pnp*			
SPA0045	I	5344900-5345100	PA4758.1	5	-	5616829-5617029	*carA*/*dapB*			P32
SPA0046	I	5439800-5439900	PA4844/*dipZ*	5	+	5710452-5710552	*ctpL*/*dipZ*			
SPA0047	I	6110800-6111000	*aspA*/PA5430	5	-	6383723-6383923	*aspA*/PA14_71670			
SPA0048	II	990900-991100	*rsmA*	4	+	4660491-4660691	*rsmA*			
SPA0049	II	1182800-1183100	*fliC*	5	+	4466831-4467131	*fliC*			
SPA0050	II	3762700-3763000	*flgM*	6	+	1784235-1784535	*flgM*			
SPA0051	II	5337600-5337800	*ftsH*	5	-	5609529-5609729	*ftsH*			
SPA0052	II	5522100-5522300	*azu*	6	-	5792869-5793769	*azu*			
SPA0053	II	5813600-5813800	PA5165	4	+	6085771-6085971	PA14_68230			
**SPA0054**	III	176100-176200	*pcaG*	15	-	174851-175160	*pcaG*			

		PAO1				PA14				
**nstSGR name[e]**	**class**	**Genomic coordinates (left-right)**	**Flanking/ Involved *loci*[a]**	**# reads[b]**	**strand[c]**	**Genomic coordinates (left-right)**	**Flanking/ Involved *loci*[a]**	**# reads[b]**	**strand[c]**	**Notes[d]**
**SPA0055**	III	719900-720200	PA0667	8	+	730790-731290	PA14_08540/ *tyrZ*			
**SPA0056**	III	781400-782200	PA0708	14	+	4898197-4898997	PA14_55150			
SPA0057	III	782900-783600	PA0711	7	+	4896798-4897497	PA14_55117			
SPA0058	III	1027800-1028000	PAO937	4	-	4623557-4623757	*yaiL*			
SPA0059	III	2113600-2114100	PA1933	5	-	3518295-3518795	PA14_39520			
SPA0060	III	3348400-3348700	PA2990	4	-	2220645-2220945	*ugpQ*			
**SPA0061**	III	3349200-3349400	*sth*	8	+	2219945-2220145	*sth*			
SPA0062	III	3570600-3570800	PA3180	2	-	2003152-2003352	*mutT*			
SPA0063	III	3617500-3617600	PA3230	4	+	1951512-1951612	PA14_22410			
SPA0064	III	3865900-3866200	PA3459	4	-	1674746-1675046	*asnB*			
SPA0065	III	4245900-4246100	PA3788	4	-	1280381-1280581	PA14_15090			
SPA0067	III	5676000-5676200	*pilQ*	4	+	5947414-5947580	*pilQ*			
Group D										
SPA0068	I	332900-333100	PA0295/*spuI*	6	-	347000-347200	PA14_03855/PA14_03860	4	-	
SPA0069	I	586800-587000	*rsmY*	205	+	596800-597000	*rsmY*	99	+	RsmY
**SPA0070**	I	706700-706900	*vfr*/PA0653	12	-	717700-717800	*vfr*/PA14_08380	4	-	
**SPA0071**	I	883400-883600	PA0805/PA0806	5	-	4773800-4773900	PA14_53830/PA14_53840	5	+	
**SPA0072**	I	901500-901900	*ssrA*	1643	-	4749700-4750100	PA14_53560/PA14_53570	1595	+	tmRNA
SPA0073	I	1205000-1205100	PA1112.1	4	-	4444700-4445000	PA14_49990/PA14_50000	7	+	sRNA645
**SPA0074**	I	1557900-1558200	PA1429/*lasR*	6	+	4086100-4086300	*lasR*/PA14_45970	8	-	
SPA0075	I	1668800-1669200	*ffs*	862		3974500-3974700	PA14_44640/PA14_44650	500	-	4.5S
SPA0076[f]	I					3975600-3975800	PA14_44640/PA14_44650	555	-	4.5S
**SPA0077**	I	2473000-2473100	*bkdR*/*bkdA1*	8	+	3159700-3159900	*bkdA1*/*bkdR*	8	-	
**SPA0078**	I	2705600-2705800	PA2421/PA2422	6	-	2937800-2937900	PA14_33370/PA14_33380	7	+	
**SPA0079**	I	3123200-3123500	PA2763/PA2764	1893	-	2450500-2450700	PA14_28350/PA14_28360	2982	+	
**SPA0080**	I	3147500-3147600	PA2789/PA2790	7	+	2426600-2426800	PA14_28030/PA14_28040	25	-	
**SPA0081**	I	3442300-3442500	PA3069/PA3070	5	-	2129000-2129100	*moxR*/PA14_24440	5	+	
SPA0082	I	3705300-3705600	*phrS*	80	-	1841700-1842000	PA14_21260	120	+	PhrS; P20
SPA0083	I	3778000-3778200	*amiL*	28	-	1768500-1768600	PA14_20550/*amiE*	3	+	AmiL
**SPA0084**	I	3958000-3958200	PA3535/PA3536	14	+	1599900-1600100	PA14_18620/PA14_18630	36	-	

		PAO1				PA14				
**nstSGR name[e]**	**class**	**Genomic coordinates (left-right)**	**Flanking/ Involved *loci*[a]**	**# reads[b]**	**strand[c]**	**Genomic coordinates (left-right)**	**Flanking/ Involved *loci*[a]**	**# reads[b]**	**strand[c]**	**Notes[d]**
**SPA0085**	I	4057500-4057700	*rsmZ*	3494	-	1500300-1500500	*rpoS*/*fdxA*	8491	+	RsmZ
**SPA0086**	I	4388800-4389000	PA3919/PA1920	11	-	1135100-1135500	PA14_13170/PA14_13190	7	+	
**SPA0087**	I	4514400-4514700	PA4033/*aqpZ*	10	+	1011600-1011900	*aqpZ*/PA14_11670	20	-	
**SPA0088**	I	4519000-4519100	PA4036/PA4037	4	-	1007000-1007300	PA14_11620/PA14_11630	4	+	
SPA0089	I	4536600-4536900	*ribC*/*ribD*	9	-	989600-989800	*ribD*/*ribC*	4	+	sRNA2315
SPA0090	I	4939100-4939300	PA4406.1	10	-	5102700-5102900	*lpxC*/*ftsZ*	3	-	72/101
SPA0091	I	4956300-4956700	*rnpB*	340	-	5119800-5120100	PA14_57460/PA14_57470	630	-	RnpB; sRNA2510
**SPA0092**	I	5308500-5309000	*crcZ*	100	+	5580500-5581300	*cbrB*/*pcnB*	120	+	CrcZ
SPA0093	I	5372500-5372600	PA4784/PA4785	10	-	5644200-5644400	PA14_63240/*yfcY*	4	-	
SPA0094	I	5884300-5884600	*ssrS*	898	+	6156500-6156800	PA14_69030/PA14_69040	767	+	6S
SPA0095	I(PAO1)/III(PA14)	1997000-1997400	*cysI*/PA1839	8	+	3637200-3637500	PA14_40740	5	-	sRNA1059
**SPA0096**	I(PAO1)/V(PA14)	3114100-3114200	PA2751/PA2752	11	-	2459700-2460900	PA14_28520	41	+	
**SPA0097**	I(PAO1)/V(PA14)	3129600-3129700	PA2771/PA2770	15	+	2444300-2444400	PA14_28250	78	-	RGP43
SPA0098	II	410900-411500	PA0367	4	+	425649-426248	PA14_04820	3	+	
SPA0099	II	564700-564900	PA0506	3	+	574700-575000	PA14_06600	6	+	
**SPA0100**	II	648600-649000	PA0588	9	-	661400-661700	*prkA*	12	-	
**SPA0101**	II	1348400-1348600	PA1244	8	-	4286000-4286200	PA14_48150	7	+	
**SPA0102**	II	3549800-3550100	*rpsA*	8	-	2024000-2024200	*rpsA*	5	+	
**SPA0103**	II	3616900-3617100	PA3229	59	+	1951900-1952200	PA14_22420	3	-	
**SPA0104**	II	3889700-3899900	*rhlI*	33	-	1651700-1651900	*rhlI*	70	+	
SPA0105	II	3892800-3893300	*rhlA*	6	-	1648400-1649100	*rhlA*	17	+	
**SPA0106**	II	4622700-4622800	PA4133	11	+	904900-905200	*ccoN*	4	-	
SPA0107	II	5067700-5067900	PA4523	4	-	5231400-5231600	PA14_58690	3	-	
SPA0108	II	5165800-5166000	PA4606	2	-	5438300-5438500	PA14_60960	2	+	
SPA0109	II	5952700-5952900	*glnK*	9	-	6226000- 6226100	*glnK*	13	-	
**SPA0110**	II	6162000-6162300	PA5473	3	+	6434900-6435100	PA14_72230	6	+	
**SPA0111**	III	182500-182700	*triC*	12	-	181400-181600	Pa14_01970	7	-	
**SPA0112**	III	410900-411500	PA0367	2	-	425649-426248	PA14_04820	5	-	
**SPA0113**	III	449000-449400	*gshB*	9	+	463700-464100	*gshB*	34	+	
**SPA0114**	III	2982700-2982900	*nuoA*	3	-	2599000-2599200	*nuoA*	11	+	

		PAO1				PA14				
**nstSGR name[e]**	**class**	**Genomic coordinates (left-right)**	**Flanking/ Involved *loci*[a]**	**# reads[b]**	**strand[c]**	**Genomic coordinates (left-right)**	**Flanking/ Involved *loci*[a]**	**# reads[b]**	**strand[c]**	**Notes[d]**
**SPA0115**	III	3119200-3119700	PA2759	33	+	2454200-2454800	PA14_28410	88	-	
**SPA0116**	III	3127700-3127900	PA2769	20	+	2446100-2446200	PA14_28290	89	-	
**SPA0117**	III	3414600-3414800	*pyrD*	5	+	2154400-2154700	*pyrD*	8	-	
**SPA0118**	III	3761800-3762100	PA3350	14	-	1785200-1785400	*flgA*	5	+	
**SPA0119**	III	5680700-5681300	*ponA*	27	-	5951200-5952700	*ponA*	11	-	
SPA0120	III	6135900-6136000	*wbpZ*	5	+	6408800-6409000	*wbpZ*	9	+	
**SPA0121**	III	6171700-6172000	PA5480	14	-	6444600-6445100	PA14_72350	4	-	
**SPA0122**	III	6183500-6183700	PA5492	36	-	6456400-6456600	*engB*	73	-	
SPA0123	V	784000-784500	PA0713	17	+	4895600-4896200	PA14_55110	12	-	
**SPA0124**	V	1538600-1539000	PA1414	44	+	4105200-4105600	PA14_46160	197	-	
SPA0125	V	3108800-3109300	PA2747	9	-	2464700-2465200	PA_28600	12	+	
SPA0126	V	3710200-3710700	PA3309	14	+	1836400-1836900	PA14_21220	10	-	
SPA0127	V	5544700-5545000	PA4940	4	-	5816100-5816300	PA14_65260	4	-	
Group E										
SPA0128	I	99747-100047	*fha1*/*tssA1*			97400-97700	PA14_00980/PA14_00990	3	-	
**SPA0129**	I	517688-518088	PA0458/PA0459			530600-531100	*emrA*/*clpA*	4	+	
SPA0130	I	674110-674210	PA0611/PA0612			686800-686900	*prtR*/PA14_07970	3	+	
**SPA0131**	I	3813773-3813973	*hasAp*/*hasD*			1725300-1725500	*hasAP*/*hasD*	5	-	
SPA0132	I	3498188-3498388	*fimV*/PA3116			2069900-2070100	PA14_23810/*fimV*	6	+	
SPA0133	I	3221081-3221181	PA2867/PA2868			2348200-2348300	PA14_26990 PA14_27000	3	-	
SPA0134	I	2966810-2967110	*icd*/*idh*			2614800-2615100	*idh*/*icd*	7	-	
**SPA0135**	I	2893791-2894091	PA2559/PA2560			2733300-2733600	PA14_31430/PA14_31440	3	+	
SPA0136	I	2341596-2341796	PA2127/*cupA1*			3301600-3301800	PA14_37060/PA14_37070	4	+	
SPA0137	I	1720609-1720709	*gltA*/*sdhC*			3922800-3922900	*sdhC*/*gltA*	4	-	
SPA0138	I	1163027-1163327	*braC*			4486600-4486900	*braC*	5	+	
SPA0139	I	5282368-5282768	PA4702/PA4703			5554300-5554700	PA14_62250/PA14_62240	8	+	
SPA0140	II	8500-8700	PA0007			8500-8700	PA14_00080	7	+	
SPA0141	II	40479-40679	PA0039			40500-40700	PA14_00480	4	+	
SPA0142	II	141786-142186	PA0122			139700-140100	PA14_01490	17	+	
**SPA0143**	II	437564-437864	*pilU*			452300-452600	*pilU*	6	+	

		PAO1				PA14				
**nstSGR name[e]**	**class**	**Genomic coordinates (left-right)**	**Flanking/ Involved *loci*[a]**	**# reads[b]**	**strand[c]**	**Genomic coordinates (left-right)**	**Flanking/ Involved *loci*[a]**	**# reads[b]**	**strand[c]**	**Notes[d]**
SPA0144	II	4507749-4507948	PA4026			1018300-1018500	PA14_11750	3	-	
**SPA0145**	II	3772836-3773036	*lecB*			1774200-1774400	*lecB*	6	-	
**SPA0146**	II	3649323-3649523	PA3261/PA3262			1897700-1897000	PA14_21830	4	+	
**SPA0147**	II	3332328-3332928	*rne*			2236400-2237000	*rne*	10	-	
SPA0148	II	2926236-2926636	*gacA*			2655300-2655700	*gacA*	4	+	
SPA0149	II	2059117-2059417	PA1888			3573000-3573300	PA14_40100	5	+	
**SPA0150**	II	1703023-1703223	*acnA*			3940300-3940500	*acnA*	5	+	
SPA0151	II	1226131-1226232	PA1134			4423700- 4423800	PA14_49720	4	+	
SPA0152	II	0939003-940203	PA0861			4711400-4711600	PA14_53140	4	-	
SPA0153	II	5755147-5756347	*estA*			6028400-6028600	*estA*	5	-	
SPA0154	II	6106977-6107377	*adhA*			6379900-6380300	*adhA*	8	+	
**SPA0155**	III	131185-131585	*colII*			129100-129500	*colII*	4	-	
**SPA0156**	III	334675-334975	*spuA*			348800-349100	*spuA*	6	-	
**SPA0157**	III	380028-380328	*ptsP*			394200-394500	*ptsP*	3	-	
SPA0158	III	4751619-4751918	*bfrA*			774900-775200	*bfrA*	6	-	
SPA0159	III	4005030-4005330	PA3573			1552800-1553100	PA14_18090	11	-	
**SPA0160**	III	3501188-3500688	*leuB*			2067200-2067500	*leuB*	3	-	
**SPA0162**	III	2911211-2910811	*alkB1*			2672600-2672800	*alkB1*	3	-	
**SPA0163**	III	2818447-2818747	PA2500			2808600-2808900	PA14_32330	3	+	
SPA0164	III	1996877-1997277	PA1838/PA1839			3637400 -3637800	PA14_40750	5	+	
**SPA0165**	III	1878304-1878604	PA1735			3756200-3756500	PA14_42100	4	+	
SPA0166	III	1558629-1558929	*lasR*			4085300 -4085600	*lasR*	10	+	
**SPA0167**	III	1265312-1264912	PA1166/*pcpS*			4383800-4384000	PA14_49330/*pcpS*	3	-	
**SPA0168**	III	1096531-1096631	*purC*			4553300-4553400	*purC*	40	+	
SPA0169	III	1068967-1069257	PA0987			4583200-4583500	PA14_51540	5	+	RGP7
SPA0170	III	5001909-5002309	PA4473			5165500-5165900	PA14_58060	4	-	
SPA0171	III	5301669-5301869	PA4722			5573600-5573800	*aspC*	4	+	
SPA0172	III	5868087-5868387	PA5212/*gcvP*			6140300-6140600	PA14_68840/*gcvP1*	7	+	
SPA0173	III	5885587-5885887	PA5229			6157800-6158100	PA14_69050	6	-	
**SPA0174**	III	5925886-5926066	*argH*			6198100-6198300	*argH*	3	-	

		PAO1				PA14				
**nstSGR name**[e]	**class**	**Genomic coordinates (left-right)**	**Flanking/ Involved *loci***[a]	**# reads**[b]	**strand**[c]	**Genomic coordinates (left-right)**	**Flanking/ Involved *loci***[a]	**# reads**[b]	**strand**[c]	**Notes**[d]
SPA0175	III	5995982-5996181	PA5325			6268900-6269100	PA14_70300	4	-	
SPA0176	III	6176689-6176989	*ampDH2*			6449600-6449900	PA14_72400	4	+	
SPA0177	III	6218466-6218866	PA5525			6491700-6492100	PA14_72890	11	-	
SPA0178	V	4632556-4632856	PA4141			895000-895300	PA14_10360	15	-	
SPA0179	V	3235659-3236359	PA2883			2333200-2333600	PA14_26780	12	-	
SPA0181	V	3108471-3108771	PA2746/PA2747			2465200-2465500	PA14_28610	5	-	
SPA0182	V	1064311-1064707	PA0981/PA0982			4585500-4585900	PA14_51570	6	-	RGP7
SPA0184	V	5169142-5169442	PA4611			5441600-5441900	PA14_61010	7	-	

[a] *Loci* (locus ID or gene name) either overlapping (class II, III and V) or flanking (class I) the nstSGRs.
[b] Total number of cDNA reads identifying the nstSGR.
[c] Upper (+) or lower (-) genomic DNA strand coincident with cDNA reads.
[d] Names of annotated *P. aeruginosa* sRNAs; Region of Genomic Plasticity (RPG) containing the nstSGR.
[e] Tested sRNAs are in boldface.
[f] In PA14 this nstSGR contains a duplication of *ffs* gene for 4.5S between *loci* PA14_44640 and PA14_44650.
