# Supplementary material for: Comparative Profiling of Pseudomonas aeruginosa Strains Reveals Differential Expression of Novel Unique and Conserved Small RNAs
Source: PLoS One. 2012 May 10;7(5):e36553. doi: 10.1371/journal.pone.0036553 (PMC3349714; doi:10.1371/journal.pone.0036553)
Supplement: Table S3 — Previously identified P. aeruginosa sRNAs found in this work. (PDF) [file pone.0036553.s006.pdf]

Table S3. Previously identified *P. aeruginosa* sRNAs found in this work.

			PAO1	PA14
**nstSGR name**	**sRNA name**	**Reference[c]**	**# reads[a]**	**# reads[a]**
Group A				
SPA0001	PhrD	[19]	95	-
Group C				
SPA0028	P5	[17]	5	-
SPA0042	P26	[17]	4	-
SPA0045	P32	[17]	5	-
Group D				
SPA0069	RsmY	[17,21]	205	99
SPA0072	tmRNA	[19]	1643	1595
SPA0073	sRNA645	[18]	4	7
SPA0075	4.5S	[18,19,50]	862	500
SPA0076[b]	4.5S	[18,19,50]	-	555
SPA0082	PhrS; P20	[17,19]	80	120
SPA0083	AmiL	[19]	28	3
SPA0085	RsmZ	[22]	3494	8491
SPA0089	sRNA2315	[18]	9	4
SPA0090	72/101	[19]	10	3
SPA0091	RnpB; sRNA2510	[17-19]	340	630
SPA0092	CrcZ	[20]	100	120
SPA0094	6S	[19,51]	898	767
SPA0095	sRNA1059	[18]	8	5
SPA0122	Spot42	[31]	36	73

[a] Total number of reads identifying nstSGR.
[b] In PA14 this nstSGR contains a duplication of *ffs* gene for 4.5S between *loci* PA14_44640 and PA14_44650.
[c] References [1]-[49] as in main text. [50] Toschka HY, Struck JC, Erdmann VA (1989) The 4.5S RNA gene from *Pseudomonas aeruginosa*. Nucleic Acids Res 17: 31-36; [51] Vogel DW, Hartmann RK, Struck JC, Ulbrich N, Erdmann VA (1987) The sequence of the 6S RNA gene of *Pseudomonas aeruginosa*. Nucleic Acids Res 15: 4583-4591.
